# Utility of the 3Di short version in the identification and diagnosis of autism in children at the Kenyan coast

**DOI:** 10.3389/fpsyt.2024.1234929

**Published:** 2024-02-29

**Authors:** Patricia Kipkemoi, Symon M. Kariuki, Joseph Gona, Felicita Wangeci Mwangi, Martha Kombe, Collins Kipkoech, Paul Murimi, William Mandy, Richard Warrington, David Skuse, Charles R.J.C. Newton, Amina Abubakar

**Affiliations:** ^1^ Neuroscience Unit, Kenya Medical Research Institute (KEMRI)-Wellcome Trust Research Programme, Kilifi, Kenya; ^2^ Complex Trait Genetics Department, Center for Neurogenomics and Cognitive Research (CNCR), Vrije Universiteit Amsterdam, Amsterdam, Netherlands; ^3^ Department of Psychiatry, University of Oxford, Warneford Hospital, Oxford, United Kingdom; ^4^ Department of Public Health, Pwani University, Kilifi, Kenya; ^5^ Department of Psychiatry, Moi Teaching and Referral Hospital, Eldoret, Kenya; ^6^ Institute for Human Development, Aga Khan University, Nairobi, Kenya; ^7^ Division of Psychology and Language Sciences, University College London (UCL) Research Department of Clinical, Educational and Health Psychology, London, United Kingdom; ^8^ Institute of Child Health, University College London (UCL), London, United Kingdom

**Keywords:** autism, diagnosis, Africa, 3Di, psychometrics, reliability

## Abstract

**Introduction:**

The precise epidemiological burden of autism is unknown because of the limited capacity to identify and diagnose the disorder in resource-constrained settings, related in part to a lack of appropriate standardised assessment tools and health care experts. We assessed the reliability, validity, and diagnostic accuracy of the Developmental Diagnostic Dimensional Interview (3Di) in a rural setting on the Kenyan coast.

**Methods:**

Using a large community survey of neurodevelopmental disorders (NDDs), we administered the 3Di to 2,110 children aged between 6 years and 9 years who screened positive or negative for any NDD and selected 242 who had specific symptoms suggestive of autism based on parental report and the screening tools for review by a child and adolescent psychiatrist. On the basis of recorded video, a multi-disciplinary team applied the Autism Diagnostic Observation Schedule to establish an autism diagnosis. Internal consistency was used to examine the reliability of the Swahili version of the 3Di, tetrachoric correlations to determine criterion validity, structural equation modelling to evaluate factorial structure and receiver operating characteristic analysis to calculate diagnostic accuracy against Diagnostic Statistical Manual of Mental Disorders (DSM) diagnosis.

**Results:**

The reliability coefficients for 3Di were excellent for the entire scale {McDonald’s omega (ω) = 0.83 [95% confidence interval (CI) 0.79–0.91]}. A higher-order three-factor DSM-IV-TR model showed an adequate fit with the model, improving greatly after retaining high-loading items and correlated items. A higher-order two-factor DSM-5 model also showed an adequate fit. There were weak to satisfactory criterion validity scores [tetrachoric rho = 0.38 (p = 0.049) and 0.59 (p = 0.014)] and good diagnostic accuracy metrics [area under the curve = 0.75 (95% CI: 0.54–0.96) and 0.61 (95% CI: 0.49–0.73] for 3Di against the DSM criteria. The 3Di had a moderate sensitivity [66.7% (95% CI: 0.22–0.96)] and a good specificity [82.5% (95% CI: 0.74–0.89)], when compared with the DSM-5. However, we observed poor sensitivity [38.9% (95% CI: 0.17–0.64)] and good specificity [83.5% (95% CI: 0.74–0.91)] against DSM-IV-TR.

**Conclusion:**

The Swahili version of the 3Di provides information on autism traits, which may be helpful for descriptive research of endophenotypes, for instance. However, for accuracy in newly diagnosed autism, it should be complemented by other tools, e.g., observational clinical judgment using the DSM criteria or assessments such as the Autism Diagnostic Observation Schedule. The construct validity of the Swahili 3Di for some domains, e.g., communication, should be explored in future studies.

## 1 Introduction

Autism has been reported to affect 1 in 100 children, and the prevalence has increased over the years, based on reports from high-income settings (1, 2). The increasing prevalence is ascribed to improved recognition of the condition over time or an epidemiological change of underlying risk factors such as prenatal, perinatal, and postnatal environmental factors (3–5).

Approximately 95% of children under the age of 5 years with developmental disabilities such as autism live in lower- and middle-income countries; however, most research is based on children with autism from high-income countries (6). There are few data on the epidemiology of autism in Africa, related to limited awareness about the condition, the complexity of diagnosis, owing to few experts such as psychiatrists and psychologists, poor health records, lack of validated and standardised assessment tools for autism, and limited investment in research on this subject (7). Available community estimates of pervasive developmental disorders (PDDs) in young children suggest that autism may be common in African populations (8–10), but these estimates need diagnostic confirmation.

There are two main diagnostic guidelines proposed for the characterisation of autism, namely, the American Psychiatric Association Diagnostic Statistical Manual of Mental Disorders (DSM) (11) and the International Statistical Classification of Diseases and Related Health Problems, 10th revision (ICD-10), created by and supported by the World Health Organisation (12). The APA developed the DSM-IV-TR criteria, which outline three domains of autism symptoms: qualitative impairment in social interaction, impaired communication, and restricted and repetitive behaviours and interests. Individuals suspected of autism must show behaviours that fall into at least two sub-domains (in the area of social reciprocity, and one sub-domain on communication and another in restricted and repetitive behaviours and interests), and these disturbances should cause clinically significant functional impairment (11). The criteria were updated in 2013; DSM-5 criteria drew on research on the domains of autism symptoms to reformulate the previously used three-dimensional model to a collapsed two-dimensional model. In the ICD-10, PDDs include childhood autism, atypical autism, Rett syndrome, Asperger’s syndrome, and childhood disintegrative disorder, all of which include 12 criteria grouped into three domains, namely, social interaction, communication, and restricted interests and behaviours, just as with the DSM-IV criteria.

Standardised tools have been adapted and validated to screen for and diagnose autism in children. The most popular ones include the Autism Diagnostic Interview–Revised (ADI-R), the Autism Diagnostic Observation Schedule (ADOS) (13), and the extended version and a short version of Developmental Diagnostic Dimensional Interview (3Di) (14). ADI-R is a semi-structured, investigator-based interview for caregivers of children and adults for whom autism is suspected. The 3Di is a parental interview comprising the three domains of social reciprocity, communication, and repetitive and stereotyped behaviour derived from the DSM-IV-TR criteria.

The short version of the 3Di (3Di-sv) is increasingly being used in different parts of the world for identification of autism (15–18) because it has 53 items (way fewer than the 112 items from the long-version scale) and takes about 45 min to administer, which is not a significant burden in challenged health care systems. The 53 items are distributed as follows: reciprocal social interaction domain (25 items), communication domain (20 items), and restricted and repetitive behaviours and interests (RRBIs) (eight items). These interviews have computerised algorithms, making scoring easier in settings where there are no trained experts; the paper-based format is also available. The 3Di-sv has excellent psychometric properties and criterion validity that compare well with those from the long version (15). However, these psychometric findings are based on studies from high-income countries such as the United Kingdom and The Netherlands and low- and middle-income countries such as Thailand and China (14, 17, 18), with none from sub-Saharan Africa. Some of these validation studies had inherent limitations, including (i) validation on children with clinically confirmed ASD, which may yield fewer false positives than in community samples; (ii) use of children with symptoms of developmental disability unrelated to ASD; and (iii) validation on definite control sample, which may inflate false negative scores. In many settings, in Africa, a clinically confirmed ASD sample meeting the 3 DSM criteria is not common. It is important to add to this body of work using community-based participants, where initial screening of ASD symptoms is carried out. Afterward, diagnostic assessments such as the 3Di or the ADOS are performed in one study.

Most autism screening and diagnostic tools are not routinely used in Africa because they are costly, are time-consuming, and require some expertise and training to administer and interpret the resultant data. Use of these scales in African populations may require adaptation and validation to explore the level of familiarity with the tests, to examine whether any modifications are needed to make them more contextually and culturally relevant, and to assess the level of expertise and training for their administration. This ensures that the tool retains its content and face validity, in addition to satisfactory psychometric properties. Fortunately, the past decade has seen an acceleration in the capacity to develop, validate, and adapt mental health assessment tools in Africa, providing promise for advancing research in autism in many resource-constrained settings of Africa (19, 20).

In view of these gaps and the underutilisation of 3Di in the assessment of autism in Africa, we used data from an epidemiological survey of neurodevelopmental disorders (NDDs) on the Kenyan coast to examine the psychometric properties, diagnostic accuracy, and measurement invariance of the short version of 3Di in this rural Kenyan setting. Specifically, we conducted a population-based study to (i) evaluate the reliability of the Swahili version of the 3Di-sv in the detection of autism in children from rural Kenyan settings; (ii) determine the factorial structure of Swahili 3Di-sv, including comparing non-nested models of the two-factor DSM-5 vs. the three-factor model derived from DSM-IV-TR; (iii) investigate the measurement invariance of the Swahili 3Di-sv in these settings; and (iv) compute the criterion validity of the Swahili 3Di-sv while comparing its scores against the diagnosis from either DSM-IV-TR or DSM-5. Given the widely recognised influence of sex in estimates of autism, we performed an analysis to investigate whether sex influenced any of these four objectives outlined above.

## 2 Materials and methods

### 2.1 Study setting

This study was carried out in a defined area, the Kilifi Health and Demographic Surveillance System (KHDSS), in Kilifi County on the Kenyan coast. The KHDSS is divided into enumeration zones, whose population is under regular surveillance for vital statistics, with the details linked to a database of admissions to Kilifi County Hospital admissions. The KHDSS has a population of ~300,000 residents, many of whom are predominantly of the Mijikenda community, the majority of whom live below the poverty line with limited formal education while practising subsistence farming and fishing (https://kemri-wellcome.org/programme/surveillance/). The health care systems in place for mental and neurodevelopmental disorders are not well-developed in Kilifi County and, more generally, in Kenya. There are no inpatient or community-based facilities for people with mental health problems, nor are there sufficient psychiatrists or psychologists in the general hospitals where care is limited to two public psychiatric outpatient clinics managed by two psychiatric nurses (21).

### 2.2 Sampling

This study was nested in a large epidemiological study that screened 11,223 children randomly selected from 28,000 children aged 6 years to 9 years within the KHDSS. The 6-year- to 9-year-old children were selected because this is when most NDDs, such as ASD, become easily recognisable in our setting and when these children can benefit from public health interventions aimed at improving school performance and quality of life. In this baseline epidemiological study, 11,223 children were successfully screened with the INCLEN NDDs screening tool (NDST) in stage I (22). There are eight autism-specific questions in the NDST; please see **Supplementary Table 1** for these questions. Approximately 2,162 children from stage I were invited for further clinical and neuropsychological assessments, including administration of the ADOS (targeted to a proportion of children screening positive in autism-specific questions) and 3Di (targeted to every child in stage 2). During piloting, NDST screened for autism with a sensitivity of 96.5% [95% confidence interval (CI): 96.1%, 96.8%] and a specificity of 80.6% (95% CI: 79.9, 81.3%) (23) and also low positive predictive values (<17.8%). There is, however, a need to follow-up screening instruments with a diagnostic instrument such as the 3Di.

### 2.3 Study design and procedures

The study was nested in a two-phase cross-sectional survey that was designed to estimate the prevalence of NDDs and to determine the risk factors of NDDs. In the first stage, NDDs were screened using the NDST questionnaire in 11,223 children aged 6 years to 9 years, randomly selected from the KHDSS (22, 24). All children who screened positive (cases) for an NDD using the NDST and those who screened negative from a randomly selected proportion of controls (N = 750) (in stage I) were invited to the epilepsy and neurodevelopment clinic to undergo psychiatric and neuropsychological tests (in stage II). Fieldworkers and clinicians interviewed parents/guardians, and we collected demographic, medical history, and socio-economic information. Some of the tools used in stage II included 3Di-sv and ADOS, which were administered by trained research assistants under the supervision of a developmental psychologist (AA), who were blinded to the child’s screening status in stage I.

These tools were translated into the local language, Swahili, through a standardised forward and back translation process as in previous studies in Kilifi (23; 25). Harmonisation of translations was done by a panel/team made up of a developmental psychologist, epidemiologist, and trained professionals (clinicians, linguists, and research assistants) fluent in English and Swahili and familiar with the local culture.

#### 2.3.1 Scoring of the paper-based developmental diagnostic dimensional interview

The scoring algorithm items coded on three-point scales—0 (no such behaviour), 1 (minimal evidence of such behaviour), and 2 (definite or persistent evidence of such behaviour)—are averaged into subscales, which are then summed up into primary scales that add up to create scores on the three domains of DSM-IV-TR. Please see **Supplementary Table 2** for more details. The scoring algorithm items are averaged by subscales, which are then summed up to create scores on the three primary DSM-IV-TR domains. The 3Di-sv includes an additional eight items on language development and age of first symptoms, which can be used to classify the PDD subtypes (14). The 2017 release of the extended 3DI was updated to reflect the DSM-5 criteria. The version used in this study, however, was based on the DSM-IV-TR criteria. However, in this study, all PDD subtypes were combined into one group, in line with the DSM-5 categorisation of autism spectrum disorders. The 3Di was administered by assessors trained by the developmental psychologist (AA).

#### 2.3.2 INCLEN neurodevelopmental screening tool

The NDST is a screening assessment of neurodevelopmental conditions developed in India (22, 24). Its use in the INCLEN program was to estimate the burden of NDDs in 2-year- to 9-year-old children in five sites in India. The tool has 39 items with responses on a four-point scale: “No, sometimes/less than half of the time, yes/most of the time, and do not know/not sure” is administered in approximately 25 min. In the original validation of the NDST, the authors reported good sensitivity and specificity. The validation study by Bitta et al. (23) also noted high sensitivity and specificity in all NDD domains and the domains combined.

#### 2.3.3 Autism diagnostic observation schedule

As part of the assessment, planned social tasks, also referred to as structured prompts, were carried out by a special education specialist (JG) who had received clinical training in the administration and interpretation of the autism symptoms as identified by ADOS. Opportunities for social interaction and communication are then observed in this standardised context. The ADOS-2 consists of four different modules based on age and expressive language level. The ADOS was performed and coded (0 for non-autism and 1 for autism) by one of the co-authors (JG) in consultation with a developmental psychologist (AA) and a paediatric neurologist (CN). Approximately 109 participants assessed by ADOS were videotaped as part of the reliability and validity checks that are part of the ADOS training process.

#### 2.3.4 DSM-IV-TR and DSM-5 criteria

Some children with potential NDDs were also assessed by video-recorded semi-structured psychiatric interviews of the ADOS. A child psychiatrist (FM), developmental psychologists (AA and PK) and developmental clinical officer (MK) reviewed ADOS videos from the subset of participants that were videotaped and gave an independent clinical diagnosis of ASD (yes - autism, autistic features, or no autism) following both the DSM-5 and DSM-IV-TR criteria.

### 2.4 Procedures and statistical analysis

Data were entered into MySQL and analysed using STATA software (version 15.1, Stata Corp LP, College Station, TX, USA), primarily the item analysis and diagnostic accuracy analysis. R statistical software (version 3.6.3) (26: https://www.r-project.org/) was used for factor analysis and reliability analysis from lavaan (27), semPlot (28), and psych (27) packages.

#### 2.4.1 Reliability and item analysis

We computed the reliability of the Swahili version of the 3Di (using the reference standards mentioned above) through the classical test or internal consistency measures, Cronbach’s alpha (α), and McDonald’s omega (ω) using the psych package for R. The threshold for Cronbach’s α and Macdonald’s ω of >0.70 was considered satisfactory according to Nunnally and Bernstein criterion (29).

#### 2.4.2 Factorial structure

Determination of the factorial structure of the Swahili version of the 3Di compared non-nested and higher-order models of the two-factor model derived from DSM-5 vs. the three-factor model derived from DSM-IV-TR using structural equation modelling. We also included two additional models, one allowing for correlated errors and the other model retaining only items with high factor loadings (>0.30). Model goodness of fit was evaluated using the root mean squared error of approximation (RMSEA), comparative fit index (CFI), and the standardised root mean residual (SRMR). RMSEA and SRMR values of <0.05 indicated a good fit, and values as high as 0.08 represented reasonable errors of approximation (30), whereas CFI and Tucker-Lewis Index (TLI) values of >0.9 were considered adequate and of 0.95 were considered excellent (31).

The DSM-IV-TR model included the three scales already seen in the 3Di: social reciprocity, communication, and RRBIs. For the DSM-5 model, the social reciprocity and communication scales were merged into one, apart from subscale C4 (imaginative play) to match with the deficits in social communication criteria noted in the DSM-5 and subscale C3 (stereotyped and repetitive language was moved to the RRBI scale to tally with the restricted and repetitive patterns of behaviour, interests, or activities).

There is evidence of the differences in scores on standard autism measures according to sex (32). To evaluate this, we performed a *post hoc* analysis of the 3Di, which included the endorsement patterns of the items according to sex, investigating the measurement invariance of the 3Di items and data. Using multi-group CFA, we tested configural, metric, and scalar invariances of the 3Di across two groups, sex (boys vs. girls), and age (6 years to 7 years, and 8 years to 9 years). We used the CFI to test the invariance of measurement after constraining factor models in the sequence of invariance tests. If the change in CFI (ΔCFI) was ≤0.01 in magnitude, then the two models were considered to have an equivalent fit (33). As 3Di-sv items are rated on an ordinal scale, diagonally weighted least-squares estimator was used to account for the 3Di-sv.

#### 2.4.3 Validity

Criterion validity was determined by computing the agreement between the Swahili version of the 3Di and the DSM criteria. This was carried out using tetrachoric correlations in STATA. Construct validity conclusions explored in a previous construct validity testing of the 3Di (34) informed matching the items for 3Di with exemplars of the DSM-IV-TR/DSM-5. In the three-factor DSM-IV-TR model, this included three factors of reciprocal social interaction, communication, and repetitive behaviour. The two-factor DSM5 model included reciprocal social interactions, communication, and restricted and repetitive behaviour.

#### 2.4.4 Diagnostic accuracy

We computed the diagnostic accuracy [sensitivity, specificity, and area under the curve (AUC) of a receiver operating characteristic (ROC) curve] of the 3Di by comparing them against the diagnosis from either DSM-IV-TR or DSM-5. We used the developer-recommended soring algorithm to get a diagnosis of autism spectrum disorder. Sensitivity is the ability of a test to classify an individual with a disorder correctly. Briefly, sensitivity was calculated as true positive/(true positive + false negative), and specificity as true negative/(true negative + false positive). Positive predictive value is described as the percentage of individuals with a positive diagnosis who have the disorder. In contrast, negative predictive value is the percentage of individuals with a negative diagnosis who actually do not have the disorder (35). The AUC for the 3Di scores against the diagnosis from DSM-IV-TR/DSM-5 was computed using non-parametric ROC approaches. We used the developer-recommended cutoff scores and scoring algorithm that requires a threshold score of 10 and above for the social reciprocity domain. Thereafter, we computed a communication score from all communication-related questions (verbal and non-verbal); a score of ≥ 8, if the child has phrase speech, or of ≥7, if the child is pre-verbal, indicates the presence of communication challenges. The presence of repetitive and stereotypical behaviour contributes to a diagnosis of atypical or typical autism. There are no clear “gold standard” or reference standards in our setting. As such, we were not able to explore different cutoff scores for the 3di-sv domains. The performance of the AUC followed the specifications discussed by Metz (36) and Hanley and McNeil (37), where a good AUC is equal to 0.90 and satisfactory AUC is equal to or above 0.65.

### 2.5 Ethics statement

The ethical approval for the study was sought from the KEMRI Scientific and Ethics Review Unit (SERU) KEMRI/SERU/CGMR-C/SSC3000. A written informed consent was obtained from the parents or guardians of all study participants prior to participation.

## 3 Results

### 3.1 General description

**Figure 1:**
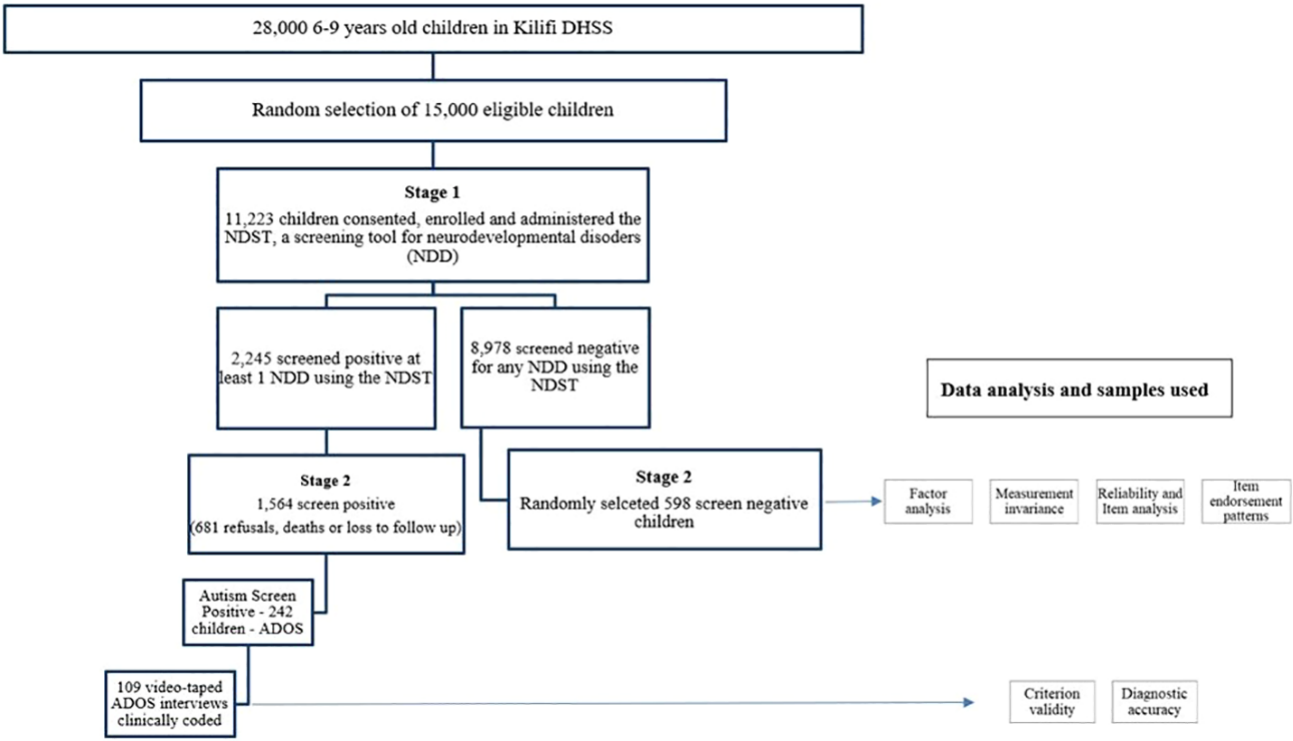
Participant flowchart of recruitment and assessment in the study.

**Figure 1** illustrates a total of 11,223 children screened, 49% of whom were girls. Of the children screened, 2,245 (20%) screened positive for at least one NDD in stage I. **Table 1** summarises the demographic characteristics of the children in the study. In total, 2,162 children were assessed in the second stage, in which 69% (N = 1,564) represents the screen-positive children and 6.7% (N = 598) represents the screen-negative children that were assessed in stage II, and there was no difference in the sexes of those who were assessed in stage II. Of those who screened positive for autism in the NDST, 242 of the children were administered the ADOS. Of these, 107 ADOS interviews were videotaped for further analysis using the DSM-5 and DSM-IV-TR criteria.

**Table 1:** Descriptive statistics of the study sample.

Characteristic	Total screened (n = 11,223)	Total stage II (n = 2,116)	Children with positive autism diagnosis	Children with negative autism diagnosis	Test statistic
Age in years					
6	1,651 (14.7%)	333 (15.7%)	16 (5.0%)	307 (95.0%)	χ2 = 4.347 p = 0.226
7	3,803 (33.9%)	711 (33.6%)	29 (4.1%)	668 (95.8%)	
8	3,636 (32.4%)	698 (33.0%)	19 (2.8%)	671 (97.2%)	
9	2,133 (19.0%)	374 (17.7%)	18 (4.9%)	353 (95.1%)	
Sex					
Male	5,646 (50.3%)	1,017 (48.1%)	41 (4.1%)	956 (95.9%)	χ2 = 0.150 p = 0.699
Female	5,577 (49.7%)	1,099 (51.9%)	41 (3.8%)	1,043 (96.2%)	

### 3.2 Factorial structure of 3Di

The adequacy of sampling (n = 2,162 participants) from the 3Di data, using the Kaiser–Meyer–Olkin (KMO) value, was explored. The KMO value was 0.50, which is borderline acceptable. A higher-order vs. simple-structure–correlated three-factor DSM-IV-TR model showed an adequate fit, with some goodness-of-fit statistics showing a poor fit (RSMEA = 0.058, SRMR = 0.070, TLI = 0.696, and CFI = 0.719) and a few items showing less than acceptable factor loadings, model parameters, degrees of freedom, and p-value (chi-square) (**Table 2**, **Supplementary Figures 2, 3**). A higher-order two-factor DSM-5 model showed a good fit when we evaluated the RMSEA and a poor fit when we evaluated the CFI and TLI (RSMEA = 0.044, SRMR = 0.063, TLI = 0.818, and CFI = 0.836) (**Supplementary Figure 4**). We can see above that the fit statistics suggest an inadequate fit of the models. If we look at the TLI and CFI, as well as the RMSEA and SRMR, the RMSEA indicates an adequate fit. As such, we carried out *post-hoc* analysis to respecify the model and re-evaluate the fit. We first evaluated the modification indices and freed error covariance constraints, correlating items with modification indices more significant than 10. This created an additional path including 13 items; we see an improvement in the DSM-IV-TR model (RMSEA = 0.018, SRMR = 0.039, TLI = 0.970, and CFI = 0.975) and the DSM-5 model (RMSEA = 0.018, SRMR = 0.039, TLI = 0.970, and CFI = 0.975) to an acceptable and good fit (**Supplementary Table 5**). The second model modification involved only retaining salient items (factor loadings equal to or greater than 0.30) in the model. This modified three-factor DSM-IV-TR model retained 15 items in the social reciprocity domain, 9 in the communication domain and 5 in the repetitive behaviour domain (**Supplementary Table 4**). Here, we also see some improvement in the model fit indices for the DSM-IV-TR model (RMSEA = 0.039, SRMR = 0.069, TLI = 0.914, and CFI = 0.921). However, for the DSM-5 model, we still see poor model fit indices (RMSEA = 0.080, SRMR = 0.125, TLI = 0.585, and CFI = 0.621). For a conceptual diagram of these models, please see **Supplementary Figures 2** to **6**.

Table 2Goodness-of-fit statistics using confirmatory factor analysis.

**Table:** 

DSM-IV-TR higher-order factor model							
	RMSEA	SRMR	TLI	CFI	Parameter	DF	P-value
DSM-IV-TR higher-order three-factor model	0.058	0.070	0.696	0.719	159	1272	<0.001
DSM-IV-TR three-factor model	0.073	0.087	0.502	0.523	109	1322	<0.001
DSM-IV-TR higher-order factor model (after correlating items with MI >10)	0.017	0.035	0.974	0.980	171	1260	<0.001
DSM-IV-TR three-factor model (keeping high loadings items)	0.039	0.069	0.914	0.921	59	347	<0.001

**Table:** 

DSM-5 higher-order factor model							
	RMSEA	SRMR	TLI	CFI	Parameter	DF	P-value
DSM-5 higher-order two-factor model	0.044	0.063	0.818	0.836	176	685	<0.001
DSM-5 two-factor model	0.080	0.108	0.415	0.444	176	738	<0.001
DSM-5 higher-order factor model (after correlating items with MI >10)	0.018	0.039	0.970	0.975	176	820	<0.001
DSM-5 two-factor model (keeping high loadings items)	0.091	0.139	0.532	0.575	44	209	<0.001

RMSEA, root mean square error of approximation (0.08 suggests an adequate fit and 0.05 suggests a good fit); CFI, comparative fit index (0.90 suggests an adequate fit and 0.95 suggests a good fit); SRMR, standardised root mean residual (0.08 suggests and adequate fit and 0.05 suggests a good fit).

### 3.3 Measurement invariance

Multi-group CFA was carried out to examine measurement invariance across sex (boys vs. girls) and age (6 years to 7 years, and 8 years to 9 years) in 2,162 participants, using both the modified three-factor DSM-IV-TR and two-factor DSM-5 model (**Table 3**) with modification indices and freed error covariance constraints. Assuming the same item factor assignment (configural invariance), the three-factor DSM-IV-TR fitted well across sex and age. Next, we tested metric invariance by assuming same-item factor assignment and additionally constraining factor loadings to equivalence across age and sex. We examined the changes in CFI where a value of ≤0.01 in magnitude means an equivalent fit for the model under review supplemented by a change in the RMSEA of ≤0.015. For comparisons greater than 0.01 (0.024) for sex and 0.01 for age, we proceeded to test scalar invariance, and, for the former, we investigated partial metric invariance by partially constraining some factor loadings. A CFI change of 0.02 was observed, which is still greater than 0.01. Therefore, we compared this partial metric invariance model to a less stringent cutoff of <0.02; we accept the partial metric invariance model. For the age category, we proceed to test metric and scalar invariance; we see a change of ≤0.01 in the CFI and a change of ≤0.015 in the RMSEA. Assuming the same item factor assignment (configural invariance), the modified two-factor DSM-5 model fitted well across sex but not across age, and, therefore, we did not proceed to test metric invariance as there was no variance on variables. When grouped into sex, we proceeded to test metric invariance, and we see a change greater than 0.01 in the CFI (0.021); we also see a change in the RMSEA of 0.009. We then proceeded to change scalar invariance, following which a change in the CFI of 0.002, but no change in the RMSEA was evident.

Table 3Measurement invariance analysis-three and two-factor models.

**Table:** 

Three-factor model						
Measurement invariance (sex)	RMSEA	SRMR	TLI	CFI	Δ CFI	Δ RMSEA
Configural invariance	0.035	0.072	0.928	0.934		
Metric invariance	0.041	0.079	0.905	0.910	0.024	−0.006
Partial metric invariance	0.040	0.078	0.908	0.913	−0.003	0.001
Scalar invariance	0.041	0.079	0.905	0.907	0.006	−0.001
Measurement invariance (age) (6–7 years and 8–9 years)	RMSEA	SRMR	TLI	CFI	Δ CFI	Δ RMSEA
Configural Invariance	0.037	0.072	0.920	0.927		
Metric Invariance	0.040	0.076	0.908	0.913	0.014	−0.003
Scalar Invariance	0.039	0.076	0.911	0.913	0.000	0.001

**Table:** 

Two-Factor Model						
Measurement invariance (sex)	RMSEA	SRMR	TLI	CFI	Δ CFI	Δ RMSEA
Configural invariance	0.008	0.045	0.995	0.995		
Metric invariance	0.017	0.051	0.974	0.977	0.021	0.009
Partial metric invariance	0.017	0.052	0.972	0.975	0.002	0.000
Scalar invariance						
Measurement invariance (age) (6–7 years and 8–9 years)	RMSEA	SRMR	TLI	CFI	Δ CFI	Δ RMSEA
Invariance	No variance in variables					

### 3.4 Reliability and item analysis

The reliability coefficients for all 3Di items were good, whether measured as McDonald’s omega [ω = 0.83 (95% CI: 0.79–0.91)] or Cronbach’ alpha [α = 0.83 (95% CI: 0.82–0.86)] values. The reliability remains very good even after stratification by sex. However, they were slightly higher for boys than girls with Cronbach’s alpha values of 0.84 (95% CI: 0.81–0.86) for boys and 0.81 (95% CI: 0.79–0.83) for girls and McDonald’s omega values of 0.81 (95% CI: 0.80–0.88) for girls and 0.87 (95% CI: 0.79–0.90) for boys (**Table 4**).

**Table 4:** Reliability statistics: sex and age.

3Di domains	Cronbach’s alpha (95% CI)			McDonald’s omega (95% CI)			Items
Domain	Overall	Male	Female	Overall	Male	Female	
Social reciprocity	0.71 (0.68–0.74)	0.69 (0.66–0.74)	0.73 (0.70–0.77)	0.71 (0.67-0.74)	0.69 (0.58–0.73)	0.73 (0.68–0.76)	25
Communication	0.65 (0.61–0.68)	0.61 (0.56–0.67)	0.67 (0.63–0.71)	0.40 (0.31-0.50)	0.22 (0.03–0.40)	0.52 (0.42–0.60)	20
RRBI	0.61 (0.50–0.66)	0.61 (0.42–0.71)	0.60 (0.48–0.70)	0.59 (0.35–0.68)	0.55 (0.25–0.70)	0.63 (0.47–0.74)	8
Domain	Overall	6–7 years	8–9 years	Overall	6–7 years	8–9 years	
Social reciprocity	0.71 (0.68–0.74)	0.71 (0.68–0.75)	0.75 (0.71–0.78)	0.71 (0.67–0.74)	0.72 (0.65–0.75)	0.71 (0.63–0.74)	25
Communication	0.65 (0.61–0.68)	0.75 (0.74–0.81)	0.82 (0.80–0.85)	0.40 (0.31–0.50)	0.36 (0.20–0.51)	0.43 (0.31–0.54)	20
RRBI	0.61 (0.50–0.66)	0.41 (0.22–0.54)	0.57 (0.44–0.67)	0.59 (0.35–0.68)	0.33 (0.17–0.60)	0.68 (0.47–0.75)	8

RRBI, restricted and repetitive behaviours and interests; CI, confidence interval.

Item factor loadings for the three-factor DSM-IV-TR model showed that there were poorly loading items (item factor loadings <0.3; **Supplementary Table 3**) in the three domains (11 in social reciprocity, 12 in communication, and 3 in RRBI). Retaining only the high-loading items in the social reciprocity and communication domain while keeping the eight items in the RRBI domain, as there are few items in the original domain, we found a slight improvement in the overall internal consistency of this model: McDonald’s omega [ω = 0.86 (95% CI: 0.84–0.87)] or Cronbach’s alpha [α = 0.85 (95% CI: 0.83–0.86)]. **Tables 4**, **5** show detailed internal consistencies in the social reciprocity and communication domain, with great improvement.

**Table 5:** Revised reliability statistics with items with high factor loadings: age and sex.

3Di domains	Cronbach’s alpha (95% CI)			McDonald’s omega (95% CI)			Items
Domain	Overall	Male	Female	Overall	Male	Female	
Social reciprocity	0.73 (0.71–0.75)	0.72 (0.68–0.75)	0.75 (0.70–0.78)	0.72 (0.69–0.75)	0.68 (0.60–0.76)	0.73 (0.69–0.78)	14
Communication	0.80 (0.79–0.82)	0.80 (0.76–0.83)	0.81 (0.78–0.83)	0.82 (0.80–0.84)	0.81 (0.79–0.84)	0.82 (0.80–0.85)	8
Domain	Overall	6–7 years	8–9 years	Overall	6–7 years	8–9 years	Items
Social reciprocity	0.71 (0.68–0.74)	0.71 (0.68–0.75)	0.75 (0.71–0.78)	0.71 (0.67–0.74)	0.69 (0.6–0.73)	0.73 (0.68–0.78)	14
Communication	0.65 (0.61–0.68)	0.75 (0.75–0.81)	0.82 (0.80–0.85)	0.40 (0.31–0.50)	0.79 (0.75–0.83)	0.84 (0.80–0.85)	8

CI, confidence interval.

### 3.5 Item endorsement patterns

**Supplementary Table 6** presents a detailed description of the item’s endorsement patterns across the general population and disaggregated data by sex. As can be seen from this table, most items had very low endorsement because the study was carried out in the community, and the expected prevalence of autistic traits in the community is low.

In the social reciprocity domain, positive endorsement of the items was almost the same in boys and girls. There was a marked significant difference in the positive endorsement pattern [boys = 512 and girls = 324, p < 0.001 for the item (Q369), which queries whether the child played imaginative games with children outside the family].

In the communication domain, positive endorsement of the items was mostly similar in boys and girls. However, there were a few questions that showed a significant difference, three from the imaginary play subscale and one from the use of context subscale.

In the RRBI domain, positive endorsement of the items was also very similar between the boys and the girls, with one question [Q743: “She has one or more over-riding particular interests (e.g., astronomy, insects, or dinosaurs), and will prefer activities involving these to anything else?”] being endorsed more by the boys (10.8%) than girls (4.7%). Please see **Supplementary Table 6** for more details on item endorsement.

### 3.6 Criterion validity

In total, there were 186 children identified as having a positive ASD diagnosis with the 3Di. The 3Di had a weak agreement when compared with clinical judgement using the DSM-IV-TR criteria (tetrachoric rho of 0.38) and a moderate agreement with the clinical judgement using the DSM-5 criteria for ASD (tetrachoric rho of 0.59) as coded from the 109 recorded ADOS videos. Please see **Table 6** for more details.

**Table 6:** Comparison of the Swahili version of the 3Di with other reference standard criteria.

	3Di, negative autism diagnosis	3Di, positive autism diagnosis	Total
DSM-IV-TR negative autism diagnosis	76 (83.5%)	15 (16.5%)	91 (100.0%)
DSM-IV-TR positive autism diagnosis	11 (61.1%)	7 (38.9%)	18 (100.0%)
Tetrachoric rho = 0.38 (p = 0.049)	87 (79.8%)	22 (20.8%)	109 (100.0%)
DSM-5 negative autism diagnosis	85(82.5%)	18 (17.5%)	103 (100.0%)
DSM-5 positive autism diagnosis	2 (33.3%)	4 (66.7%)	6 (5100.0%)
Tetrachoric rho = −0.59 (p = 0.015)	87 (79.8%)	22 (20.2%)	109 (100.0%)

Dx, diagnosis; RRBI, restricted and repetitive behaviours and interests; CI, confidence intervals.

### 3.7 Diagnostic accuracy

The 3Di showed a moderate sensitivity and a good specificity when compared to clinical judgement using the DSM-5 criteria [66.7% (95% CI: 0.22–0.96) and 82.5% (95% CI: 0.74–0.89), respectively], and, with DSM-IV-TR, we observed a poor sensitivity [38.9% (95% CI: 0.17–0.64)] and a good specificity [83.5% (95% CI: 0.74–0.91)] (**Table 7**). The AUC for ROC for 3Di discriminating against DSM-5 was satisfactory [0.75 (95% CI: 0.54–0.96)] and acceptable against DSM-IV-TR [0.61 (95% CI: 0.49–0.73)].

**Table 7:** Sensitivity and specificity analysis: 3Di and the DSM as the reference standards.

DSM-5 reference standard					
	Sensitivity (95% CI)	Specificity (95% CI)	Positive predictive value (95% CI)	Negative predictive value (95% CI)	Area under the curve (95% CI)
3Di	66.7% (0.22–0.96)	82.5% (0.74–0.89)	18.2% (0.05–0.40)	97.7% (0.92–0.99)	0.75 (0.54–0.96)
DSM-IV-TR reference standard					
3Di	38.9% (0.17–0.64)	83.5% (0.974–0.91)	31.8% (0.14–0.55)	87.4% (0.79–0.95)	0.61 (0.49–0.73)

## 4 Discussion

Reliability findings from both Cronbach’s alpha and McDonald’s omega show that the Swahili version of the 3Di has good internal consistency overall, indicating that it is reliable for the identification of autism traits, particularly in children with established autism diagnosis.

Still, the weak to moderate agreement scores for criterion validity and the low to moderate sensitivity scores caution against its use in making new autism diagnoses in the community, pointing to the limits that only parental interviews may have in the diagnosis of autism without complementing reference standard tools such as clinical assessment with the DSM and observational assessments such as the ADOS. In ideal settings, a multidisciplinary team comprising a combination of assessments would yield the most robust and accurate diagnosis (38).

Factor analysis further identified items that loaded poorly onto the factors. The high-loading items could represent a set of items that are useful for screening. Configural, metric, and scalar invariance parameters for 3Di were similar between boys and girls, although endorsement or response to some items differed slightly between the sexes. Meanwhile, criterion and diagnostic/discriminant validity for 3Di were poor when compared to the DSM criteria.

### 4.1 Reliability of the 3Di

The items from the Swahili version of the 3Di are reliable in mapping out autism traits in these settings in that they were excellent whether measured by Cronbach’ alpha or McDonald’s omega values; however, with the very low sensitivity scores, the 3Di alone is not reliable in the identification of autism in our setting. There were low factor loadings in a number of the items in each domain, most notably in the communication domain. The reliability measures for the social reciprocity and communication domains remained acceptable, with the RRBI domain having lower reliability scores. Reliability coefficients are dependent on the number of items as well as the covariance between them, which may have inevitably been affected by the few items in the RRBI domain. These reliability findings are similar to those of other settings in high-income countries (14, 17), albeit some measured internal consistency from Cronbach’s alpha rather than McDonald‘s omega values. Once the low-loading items (<0.30) were removed from the social reciprocity and communication domains, we saw a notable improvement in internal consistency. The high-loading items may be considered part of screening measures in the community, and these items appeared to be better correlated with the 3Di domains.

### 4.2 Factorial structure of the 3Di and measurement invariance

The excellent fitting for the three-factor is expected because 3Di development was mainly based on the diagnostic criteria from the DSM-IV-TR, with DSM-5 being developed more recently. The acceptable to excellent fit for the DSM-5 suggests that some items from 3Di can fit perfectly well into the autism spectrum disorder archetype suggested by the DSM, with a future focus on those items in the DSM-5 that are missing from the 3Di. Previous construct validity work with experts identified “hyper-activity and hypoactivity” as aspects of autism spectrum disorder that are under-represented in the 3Di and imitation of play subscales as those in the 3Di but not in the DSM-5 (18). Future work is needed to do more harmonisation of items between these scales. In addition, to fit indices, multigroup CFA models evaluated measurement invariance across age and sex and found that the 3Di can be generalised across these two groups.

### 4.3 Criterion validity and diagnostic accuracy

We noted that the Swahili version of the 3Di had weak to moderate agreement from the tetrachoric rho coefficients when compared with the DSM-IV-TR and DSM-5 criteria for autism. The DSM scoring was based on the prompts and incidental observations from the ADOS, which presents a limitation in the evaluation of the DSM as the reference standard. The agreement results are consistent with those for sensitivity and specificity, whereby the former is low to moderate, and the latter is high. The AUC score was sufficient to be good, thereby showing the acceptable discriminative ability of 3Di. These findings suggest that the parental interview format of the 3Di is an adequate measure in the identification of autism and would perform even better when applied in conjunction with an observational tool or clinical judgement criteria for a more robust diagnosis.

These 3Di findings are somewhat contrary to what was found in other studies. For example, a Thai study that investigated the psychometric properties of the 3Di found overall good sensitivity and specificity of 86.2% and 80.9% for the social reciprocity domain, 85.7% and 73.5% for the communication domain, and 66.7% and 80.9% for the repetitive behaviour domain, respectively (16). Another study carried out in Hong Kong (17) found an excellent sensitivity and a fair specificity (0.95 and 0.77, respectively), as were the findings of a study done in The Netherlands (18). However, it is worth noting that these studies cannot be directly compared with our study because (i) they are from different cultural and contextual settings than ours; (ii) more robust diagnostic information was available for the other studies from sources such as electronic health records and multidisciplinary diagnostic procedures; (iii) used different unrepresentative samples, e.g., clinical samples of confirmed autism, and screened out controls for conditions unrelated to autism; and (iv) none tested the performance of the tools in a nested epidemiological survey like ours.

### 4.4 Sex considerations in reliability and validity

We also noted that the Swahili version of the 3Di diagnoses of autism both identified boys and girls with nearly the same frequency, and most reliability and validity measures were similar between the sexes. Current autism criteria are mainly based on research with an over-representation of boys and men and do not fully consider the presentation of autism in girls, so evaluating reliability statistics and diagnostic validity according to sex is justified in this and other studies in the literature (39, 40). Cognitive interviews and construct validity studies could clarify this finding, given that current literature consensus is that more boys than girls are diagnosed with autism (41).

### 4.5 Cultural and contextual influences in autism diagnosis

It is possible that the social communication domain in autism symptomatology is sensitive to cultural differences. A common example cited in the literature is the use and quality of eye contact as one of the features in autism diagnosis varying across different communities (42). In some African settings, such as Kilifi, there are differences in developmental expectations in the social communication domain, particularly with what may be construed as appropriate initiation of conversation or interactions, back-and-forth interactions between adult caregivers and children. This may influence what caregivers would report as developmental concerns (43). It may be likely that these cultural norms influence how children relate with others in the community, leading to lower performance of reliability scores. Even when there is equivalence in constructs across cultures, there may also be differences in individual familiarity with these constructs as well (44).

Another challenge tied closely with this is the pitfalls in the translation and back-translation process; as thorough as this process can be carried out, it may have some implications for how caregivers and respondents understand these constructs. We also had the 3Di administered by newly trained fieldworkers and clinicians who may not have been able to elucidate autism symptoms during the administration of the 3Di. This may have some implications for subsequent training and supervision norms as we work toward the validation of more globally acceptable tools for varied cultural settings. There is also room to involve caregivers, individuals with autistism, and community members at large, particularly in how they understand, conceptualise, and identify various neurodevelopmental symptoms of autism (42).

Early identification and referral to care have impacts on developmental prognosis and outcomes. Further examination of the potential contributions of these cultural and contextual factors is crucial as we work on enhancing the sensitivity and specificity of diagnostic tools such as the 3Di.

### 4.6 Importance and implications for future studies

Autism symptoms are apparent in the early developmental period; the path to early intervention begins with access to valid and reliable tools suitable for use in the specific cultural setting. The Lancet Commission very rightly discussed the stepped-care approach for interventions, whereby the least-resource-intensive management strategies are offered first and, after that, followed by more specialist-driven interventions if needed (6). In the same vein, a stepped-identification approach is recommended as beneficial in low-resource settings such as Kilifi, whereby diagnostic tools such as the 3Di would be more trained-clinician driven after identification and screening by community health volunteers and primary health care workers in dispensaries in the community. With the strides made with Kenya’s disability mainstreaming through entities such as the National Council of Persons with Disabilities, established by the Government of Kenya in 2007, a diagnosis of autism, particularly in children with higher support needs, could aid in receiving of support from government entities, including subsidised schooling costs and grants awarded to parental support groups.

The translation and adaptation process of the Swahili version of the 3Di-sv was done following the recommended guidelines. However, we do note that there are a few questions that do have what linguists term “double negatives,” which may not translate as efficiently in Kiswahili and Kigiriama. There are also a few questions that have been re-worded since the current use of the 3Di-sv in an ongoing study here in Kilifi. For example, a question that previously read: “Does he/she seldom or never look at the person he/she is talking to (or otherwise communicating with), seeming actively to avoid eye contact?” was re-worded to “Does he/she actively avoid eye contact with the person he/she is communicating with?” This may improve their translation to languages used here in Kilifi and, therefore, a clearer understanding of the questions, which, in turn, leads to clearer responses. More in-depth training is also given to the administrators of the 3Di, who are now clinical officers or research nurses.

One of the things that we considered as we evaluated the results is the influence of different contexts and respondents in the different assessments used in the study. The 3Di is a parental interview tool, and the ADOS is an observation-based measure administered by a trained assessor. Clinical judgement was coded using the DSM criteria by watching videotaped ADOS assessments. The possible influence of the environmental context of the assessments has been studied, and it has been found that it may have an impact on the expression of ASD behaviour in children (45). The fact that we evaluated a proportion of videos of the ADOS to give clinical judgement on whether the child had ASD may have given us a limited view of the child’s behaviour.

The Lancet Commission acknowledges the importance of a multidisciplinary approach to the screening, diagnostic, and care process, especially on history taking, clinical observation, and current functioning in a number of contexts (6). The screening outputs of 3Di provide important information in the potential matching of interventions that may be helpful to challenges facing children with developmental disabilities such as autism and their life trajectories, notably missing in many resource-constrained settings, including the African continent.

### 4.7 Strengths and limitations

This is among the few studies to examine the reliability and validity of autism assessment tools in Africa. We nested the study in a community sample, which is free from Berkson’s or severity bias, and administered these tools following an initial screening of NDDs. The analysis approach considered both classical and modern test theories, which complemented each other very well. Another set of limitations was the few samples reviewed on DSM criteria, which included a review of these interactions from the videos of the administration of the ADOS rather than clinical observation. There is also a lack of test-retest and interrater reliability studies. These findings are only applicable in our rural settings or similar settings.

### 4.8 Conclusion

The Swahili version of the 3Di-sv in our study is reliable for use in persons with established autism to rule out false positives but does not possess more than adequate diagnostic accuracy to make new diagnoses for autism in a community sample of children in Kilifi, Kenya. This points to the challenges and limitations that may pose by using only a parental interview in the diagnosis of autism. The findings also warrant a more in-depth look at the 3Di and how the items are understood in our context, particularly those in the communication domain. Meanwhile, the 3Di-sv can be used for mapping autism symptoms in the community and thereafter following up with a confirmatory diagnostic assessment that includes a clinical observational assessment. In future, we hope to conduct cognitive interviews and construct validity analysis on the 3Di on ongoing studies to evaluate the changes to the tool, how the community understands autism, and whether there is a significant change in the diagnosis of autism.

## Data availability statement

The raw data supporting the conclusions of this article will be made available by the authors, without undue reservation. Curated data for this manuscript, deposited to the Harvard Dataverse. Kipkemoi, Patricia; Kipkoech, collins, 2024, "Replication Data for: Utility of the 3Di short version in the identification and diagnosis of autism in children at the Kenyan Coast", https://doi.org/10.7910/DVN/5NFNLA, Harvard Dataverse, V1, UNF:6:Q+OUtigkFVhcPnGiPpo3XQ== [fileUNF].

## Ethics statement

The studies involving humans were approved by the KEMRI Scientific and Ethics Review Unit (SERU) KEMRI/SERU/CGMR-C/SSC3000. The studies were conducted in accordance with the local legislation and institutional requirements. Written informed consent for participation in this study was provided by the participants’ legal guardians/next of kin.

## Author contributions

SK, CN, and AA contributed to the conception and design of the study. JG and MK were involved in data collection. PM, CK, and PK organised the database. PK and PM performed the statistical analysis. PK wrote the first draft of the manuscript. All authors contributed to the article and approved the submitted version.
