# Supplementary material for: Utility of the 3Di short version in the identification and diagnosis of autism in children at the Kenyan coast
Source: Front Psychiatry. 2024 Feb 29;15:1234929. doi: 10.3389/fpsyt.2024.1234929 (PMC10937349; doi:10.3389/fpsyt.2024.1234929)
Supplement: Supplementary file 1 [file DataSheet_1.docx]

Supplementary Material

# Utility of the 3Di short version in the identification and diagnosis of autism in children at the Kenyan Coast

**Patricia Kipkemoi* Symon M Kariuki, Joseph Gona, Felicita Wangeci Mwangi, Martha Kombe, Paul Mwangi, Will Mandy, Richard Warrington, David Skuse, Charles RJC Newton, Amina Abubakar**

*** Correspondence:** Patricia Kipkemoi, pkipkemoi@kemri-wellcome.org

## Supplementary Figures and Tables

**Supplementary Figure 1.** Participant flowchart of recruitment and assessments during the study period

**Supplementary Table 1: 3Di-sv Scales and Subscales**

| Scales and their subscales | | | | Subscale questions |
| --- | --- | --- | --- | --- |
| Social reciprocity | B1 | Use of non-verbal social cues | a | 709,710,248 |
|  |  |  | b | 249,251,252 |
|  |  |  | c | 260,261,264 |
|  | B2 | Peer and sibling relationships | a | 369 |
|  |  |  | b | 349 |
|  |  |  | c | 717,237 |
|  |  |  | d | 655,347 |
|  | B3 | Shared enjoyment | a | 303 |
|  |  |  | b | 299 |
|  |  |  | c | -304 |
|  | B4 | Emotional Reciprocity | a | 309 |
|  |  |  | b | -743 |
|  |  |  | c | 706 |
|  |  |  | d | 269 |
|  |  |  | e | 223,624,224 |
| Communication | C1 | Use of conventional gestures | a | 737 |
|  |  |  | b | 279,280,282 |
|  |  |  | c | 742 |
|  |  |  | d | 285 |
|  | C2 | Conversational Interchange | a | 679,744 |
|  |  |  | b | 705,747 |
|  | C3 | Stereotyped, repetitive or idiosyncratic speech | a | 695,696 |
|  |  |  | b | 702,703 |
|  |  |  | c | 675 |
|  |  |  | d | 749 |
|  | C4 | Imaginative play | a | 751 |
|  |  |  | b | 338,339 |
|  |  |  | c | 331 |
| Restricted, Repetitive Behaviours and Interests (RRBI) | D1 | Circumscribed interests | a | 723 |
|  |  |  | b | 754 |
|  | D2 | Ritualistic Behaviour | a | 750 |
|  |  |  | b | 756 |
|  | D3 | Simple and complex mannerisms | a | 766 |
|  |  |  | b | 767 |
|  | D4 | Non-functional use of objects | a | 755 |
|  |  |  | b | 757 |

**Supplementary Table 2: Item Factor Loadings**

1. *DSM-IV-TR*

| Domain | Subscale | Sub-subscale | Q-id | Factor loading |
| --- | --- | --- | --- | --- |
| Social Reciprocity | 1  Use of non-verbal social cues | a | **Q709** | **0.423** |
|  |  |  | **Q710** | **0.673** |
|  |  |  | Q248 | 0.154 |
|  |  | b | **Q249** | **0.375** |
|  |  |  | **Q251** | **0.434** |
|  |  |  | **Q252** | **0.441** |
|  |  | c | Q260 | -0.113 |
|  |  |  | Q261 | 0.146 |
|  |  |  | **Q264** | **0.303** |
|  | 2  Peer and sibling relationships | a | Q369 | 0.028 |
|  |  | b | Q349 | 0.010 |
|  |  | c | **Q717** | **0.503** |
|  |  |  | **Q237** | **0.590** |
|  |  | d | **Q655** | **0.346** |
|  |  |  | Q347 | 0.029 |
|  | 3  Shared enjoyment | a | **Q303** | **0.316** |
|  |  | b | Q299 | -0.165 |
|  |  | c | **Q304** | **0.303** |
|  | 4  Emotional reciprocity | a | **Q309** | **0.299** |
|  |  | b | Q743 | -0.198 |
|  |  | c | **Q706** | **0.445** |
|  |  | d | **Q269** | **0.497** |
|  |  | e | Q223 | 0.282 |
|  |  |  | Q624 | 0.270 |
|  |  |  | Q224 | 0.209 |
| Communication | Use of conventional gestures | a | Q737 | 0.050 |
|  |  | b | Q279 | 0.260 |
|  |  |  | Q280 | 0.061 |
|  |  |  | Q282 | 0.058 |
|  |  | c | Q742 | -0.064 |
|  |  | d | Q285 | -0.187 |
|  | Conversational interchange | a | **Q679** | **0.584** |
|  |  |  | Q744 | 0.147 |
|  |  | b | **Q705** | **0.723** |
|  |  |  | Q747 | 0.058 |
|  | Stereotyped, repetitive, idiosyncratic speech | a | **Q695** | **0.406** |
|  |  |  | **Q696** | **0.608** |
|  |  | b | **Q702** | **0.675** |
|  |  |  | **Q703** | **0.589** |
|  |  | c | **Q675** | **0.483** |
|  |  | d | **Q749** | **0.431** |
|  | Imaginative play | a | Q751 | 0.040 |
|  |  | b | Q338 | -0.035 |
|  |  |  | Q339 | 0.026 |
|  |  | c | Q331 | -0.058 |
| RRBI | 1  Circumscribed interests | **a** | **Q723** | **0.321** |
|  |  | **b** | **Q754** | **0.468** |
|  | 2  Ritualistic behaviour | **a** | **Q750** | **0.632** |
|  |  | **b** | **Q756** | **0.536** |
|  | 3  Simple and complex mannerisms | a | Q766 | 0.268 |
|  |  | b | **Q767** | **0.306** |
|  | 4  Non-functional use of objects | a | Q755 | 0.240 |
|  |  | b | Q757 | 0.241 |

Note: Items with factor loadings above 0.30 are highlighted in bold.

1. *DSM-5*

| Domain | Subscale | Sub-subscale | Q-id | Factor loading |
| --- | --- | --- | --- | --- |
| Social Reciprocity | 1  Use of non-verbal social cues | a | Q709 | 0.250 |
|  |  |  | **Q710** | **0.332** |
|  |  |  | **Q248** | **0.307** |
|  |  | b | **Q249** | **0.553** |
|  |  |  | **Q251** | **0.531** |
|  |  |  | **Q252** | **0.596** |
|  |  | c | Q260 | 0.050 |
|  |  |  | Q261 | 0.208 |
|  |  |  | **Q264** | **0.350** |
|  | 2  Peer and sibling relationships | a | Q369 | 0.218 |
|  |  | b | Q349 | 0.233 |
|  |  | c | Q717 | 0.284 |
|  |  |  | **Q237** | **0.307** |
|  |  | d | Q655 | 0.104 |
|  |  |  | Q347 | 0.161 |
|  | 3  Shared enjoyment | a | **Q303** | **0.529** |
|  |  | b | Q299 | -0.103 |
|  |  | c | **Q304** | **0.424** |
|  | 4  Emotional reciprocity | a | **Q309** | **0.386** |
|  |  | b | Q743 | -0.074 |
|  |  | c | **Q706** | **0.329** |
|  |  | d | Q269 | 0.193 |
|  |  | e | **Q223** | **0.405** |
|  |  |  | **Q624** | **0.434** |
|  |  |  | **Q224** | **0.412** |
| Communication | Use of conventional gestures | a | Q737 | 0.246 |
|  |  |  | Q280 | 0.241 |
|  |  |  | Q282 | 0.247 |
|  |  | c | Q742 | 0.244 |
|  |  | d | Q285 | 0.105 |
|  | Conversational interchange | a | Q679 | 0.192 |
| RRBI | 1  Circumscribed interests | **a** | Q723 |  |
|  |  | **b** | **Q754** | **0.334** |
|  | 2  Ritualistic behaviour | **a** | **Q750** | **0.543** |
|  |  | **b** | Q756 | 0.270 |
|  | 3  Simple and complex mannerisms | a | Q766 | 0.285 |
|  | 5  Stereotyped, repetitive, idiosyncratic speech | a | **Q695** | **0.523** |
|  |  |  | **Q696** | **0.691** |
|  |  | b | **Q702** | **0.602** |
|  |  |  | **Q703** | **0.598** |
|  |  | c | **Q675** | **0.475** |
|  |  | d | **Q749** | **0.537** |

Note: Items with factor loadings above 0.30 are highlighted in bold.

**Supplementary Table 3: Endorsement Patterns on the 3Di**

**Social Reciprocity – Endorsed Response**

|  | Question | Male | Female | Total | P-value |
| --- | --- | --- | --- | --- | --- |
| Q709 | She seldom or never looks at the person she is talking to (or otherwise communicating with), seeming actively to avoid eye contact? | 171 (15.89%) | 158 (15.93%) | 329 (15.91%) | 0.983 |
| Q710 | She tends to look away from the person she is talking to (or otherwise communicating with), seeming inattentive or preoccupied? | 189 (17.57%) | 177 (17.93%) | 366 (17.7%) | 0.827 |
| Q248 | If you wanted to get her attention across a crowded room, could you catch her eye? | 498 (46.46%) | 430 (43.48%) | 928 (48.0%) | 0.175 |
| Q249 | When she is approaching someone to get them to do something or to talk to them, does she smile in greeting? | 351 (32.77%) | 270 (27.36%) | 621 (30.2%) | 0.007 |
| Q251 | When she first sees you when you've been apart for a little while (when she comes home from school or you return from work, for example), does she smile in greeting? | 195 (18.14%) | 144 (14.53%) | 339 (16.4%) | 0.027 |
| Q252 | When meeting someone she knows well outside the home, does she smile in greeting? | 242 (22.53%) | 195 (19.78%) | 437 (21.2%) | 0.126 |
| Q260 | Does she spontaneously and appropriately look - surprised? | 315 (29.55%) | 253 (25.66%) | 568 (27.7%) | 0.049 |
| Q261 | Does she spontaneously and appropriately look - guilty? | 292 (27.16%) | 251 (25.33%) | 543 (26.3%) | 0.344 |
| Q264 | Does she spontaneously and appropriately look - embarrassed? | 320 (29.88%) | 247 (25.08%) | 567 (27.6%) | 0.015 |
| Q369 | Did she ever play imaginative games (including role-playing games such as dressing up) with children outside the family? | 512 (47.81%) | 324 (32.69%) | 836 (40.5%) | < 0.001 |
| Q349 | Does she invite other children to play at her home? | 365 (33.95%) | 351 (35.60) | 716 (34.7%) | 0.433 |
| Q717 | She is perceived as odd by other children and actively avoided by them. | 83 (7.74%) | 76 (7.70%) | 159 (7.7%) | 0.971 |
| Q237 | She is regarded by other people in general as rude. | 278 (25.88%) | 298 (30.10%) | 576 (27.9%) | 0.033 |
| Q655 | When engaged in an activity with other children, does She insist rigidly that they do what she says? | 427 (39.87%) | 425 (42.97%) | 852 (41.4%) | 0.153 |
| Q347 | Does she get invited to other children's houses? | 540 (50.23%) | 549 (55.57%) | 1089 (47.2%) | 0.015 |
| Q303 | Would she come to show you something she had just found, or made, or tell you about something she had just learned, or want you to share in some other event or activity she was enjoying? | 449 (41.73%) | 387 (39.01%) | 836 (40.4%) | 0.209 |
| Q299 | Does she spontaneously offer to share food treats with friends from outside the family? | 364 (33.83%) | 328 (33.10%) | 692 (33.5%) | 0.725 |
| Q304 | Does she appear to join in with the spirit of other people's pleasure or excitement? For example, would she share in the excitement of a special occasion such as Christmas? | 379 (35.19%) | 370 (37.49%) | 749 (36.3%) | 0.278 |
| Q309 | Would she realise you were upset only if it was really obvious, for example if you were actually crying, or really angry? | 156 (14.48%) | 134 (13.55%) | 290 (14.0%) | 0.541 |
| Q743 | Before they can convey their needs in words, children may drag you to what they want or direct your hand to something they can't reach. Did she do things like this, BUT with no exchange of looks and smiles, so that you felt like a tool? (Reverse scored) | 34 (3.16%) | 38 (3.89%) | 72 (3.5%) | 0.374 |
| Q706 | She seldom or never starts up a conversation, and doesn't volunteer information about what has happened? | 146 (13.66%) | 133 (13.48%) | 279 (13.6%) | 0.904 |
| Q269 | Does she sometimes laugh or smile in situations that most people would find anything but funny (e.g. somebody falling painfully in the street)? | 296 (27.82%) | 290 (29.35%) | 586 (28.6%) | 0.442 |
| Q223 | She can tell how you (as opposed to someone from outside her home) are feeling from your tone of voice? | 101 (9.40%) | 97 (9.83%) | 198 (9.6%) | 0.744 |
| Q624 | She can tell how an adult from outside her family is feeling from their tone of voice? | 161 (14.98%) | 164 (16.67%) | 325 (15.8%) | 0.293 |
| Q224 | She can tell how you (as opposed to someone from outside her home) are feeling from your facial expression? | 209 (19.48%) | 165 (16.75%) | 374 (18.2%) | 0.109 |

**Communication – Endorsed Response**

| Item | Question | Male | Female | Total | P-value |
| --- | --- | --- | --- | --- | --- |
| Q737 | If I wanted you to share my interest in a distant object, I might look at it in an obvious way, point, then look back at you, hoping to see your moment of recognition, and perhaps find you giving me a look of understanding. Does She do this? | 703 (65.2) | 654 (66.1%) | 1357 (65.6%) | 0.641 |
| Q279 | Does She - clap someone to mean 'well done'? [Interviewer: you may need to explain that you are not talking about clapping as one of a crowd] | 169 (15.68%) | 137 (13.82%) | 306 (14.8%) | 0.236 |
| Q280 | Does She - put a finger to her lips to mean 'Shhh!' | 506 (46.90%) | 460 (46.37%) | 966 (46.6%) | 0.811 |
| Q282 | Does She - beckon to someone to come closer? | 12 (16.67%) | 11 (13.75%) | 23 (15.1%) | 0.659 |
| Q742 | Does She nod her head to show that she is listening and to help maintain the flow of conversation? | 740 (68.77%) | 650 (65.66%) | 1390 (67.3%) | 0.131 |
| Q285 | In the same way, does she shake her head deliberately to mean 'No'? | 710 (66.05%) | 625 (63.00%) | 1335 (64.6%) | 0.148 |
| Q679 | Does she talk repetitively about things that no-one is interested in? | 183 (17.13%) | 172 (17.48%) | 2,052 (100%) | 0.837 |
| Q744 | Before they have speech, others can follow, infants make sounds just to be sociable. They may have a ‘conversation’ with a parent, which is really just turn-taking in making sounds. Did She do that? | 249 (23.94%) | 240 (25.24%) | 1,991 (100%) | 0.503 |
| Q705 | She ignores conversational cues from others? (e.g. if asked "What are you making?" she might continue working as if nothing had happened) | 324 (30.25%) | 295 (30.07%) | 2,052 (100%) | 0.929 |
| Q747 | When She had acquired phrase speech, did she make small talk? That is, chat with you just to be sociable, rather than to make her needs known. [Possible prompt: What about just before she started school, did she do it at that age?] | 773 (72.72%) | 710 (72.38%) | 2,044 (100%) | 0.862 |
| Q695 | Does she have favourite phrases, sentences or longer sequences which she will use a great deal, sometimes inappropriately? | 68 (6.40%) | 76 (7.76%) | 2,042 (100%) | 0.233 |
| Q696 | Does She sometimes say things she does not fully understand? | 167 (15.68%) | 152 (15.49%) | 2,046 (100%) | 0.908 |
| Q702 | Does She get into trouble because she doesn't always understand the rules of polite behaviour? | 301 (28.03) | 221 (22.35%) | 2,063 (100%) | 0.003 |
| Q703 | Did she ever make embarrassing or tactless remarks about people - perhaps saying within their hearing that someone was very fat, or ugly? | 157 (14.71%) | 137 (13.88%) | 2,054 (100%) | 0.590 |
| Q675 | When talking about other people, she sometimes makes mistakes with simple words like 'he' and she' , or 'we' and ‘they', muddling them up and using them the wrong way round? | 193 (18.11%) | 186 (18.90%) | 2,050 | 0.642 |
| Q749 | Did She ever go through a phase of using words, with little or no meaning to others, which she seemed to have made up herself? For example, she might refer to any game she was playing as ‘noob’ or repeat ‘lungdimmer’ for her own amusement. | 75 (7.03%) | 96 (9.78%) | 2,049 | 0.025 |
| Q751 | Did She go through a phase of pretending to do everyday things you, or other members of the family, did? [The emphasis here is on the element of pretense, as opposed to "mechanical" copying] | 474 (44.05%) | 287 (29.08%) | 2,063 | < 0.001 |
| Q338 | Has she ever played games with several figures or animals and these toys appeared to be talking to one another? | 731 (68.06%) | 486 (49.24%) | 2,061 (100%) | < 0.001 |
| Q339 | Has She ever made (or looked like she was making) a running commentary on the actions of her play figures or animals? | 761 (71.06%) | 565 (57.48%) | 2,054 (100%) | < 0.001 |
| Q331 | In early childhood did she join in games involving larger groups which require cooperation, such as Pass the Parcel, or Musical Chairs, or Hide and Seek? | 412 (38.29%) | 363 (36.85%) | 2,061 (100%) | 0.501 |

**Restricted, Repetitive Behaviours and Interests – Items endorsed**

| Item | Question | Male | Female | Total | P-value |
| --- | --- | --- | --- | --- | --- |
| Q723 | She has one or more over-riding particular interests (e.g. astronomy, insects or dinosaurs), and will prefer activities involving these to anything else? | 115 (10.8%) | 47 (4.8%) | 162 (7.9%) | < 0.001 |
| Q754 | Did she ever have a preoccupation with something odd or even bizarre which would NOT be of interest to most children? | 27 (2.5%) | 17 (1.7%) | 44 (2.1%) | 0.217 |
| Q750 | Did she go through a phase of endlessly and exactly repeating a word or phrase? | 47 (4.4%) | 35 (3.6%) | 82 (4.0%) | 0.332 |
| Q756 | Was there a time when She had to do things, or have you do things, in some precise yet odd sequence? [Examples: insistence on a way of getting dressed, eating foods in a specific order or pattern, or a long and complicated bedtime ritual.] | 105 (9.8%) | 98 (9.9%) | 203 (9.8%) | 0.897 |
| Q766 | Has She ever shown any hand or finger mannerisms (e.g. hand flapping or flicking fingers before her eyes) when excited or distressed? [Were these behaviours so all-absorbing that you had to discourage them? Did having to stop cause distress?] | 15 (1.4%) | 14 (1.4%) | 29 (1.4%) | 0.967 |
| Q767 | Has she ever had any other whole-body movements, or more complex mannerisms, such as spinning round, bouncing on toes, or jumping up and down on the spot? [Were these behaviours so all-absorbing that you had to discourage them?] | 10 (0.9%) | 7 (0.7%) | 17 (0.8%) | 0.577 |
| Q755 | When playing, has She ever tended to organise her toys, such as cars or bricks or animals, in lines or other patterns based upon size, colour or kind, rather than playing more imaginatively? | 39 (3.6%) | 30 (3.0%) | 69 (3.3%) | 0.451 |
| Q757 | In Her later years was there any sign of infant-like interests in taste (e.g. licking things), touch or smell (e.g. of people)? [Interviewer: choose "not applicable" if child under 3] | 21 (1.9%) | 17 (1.7%) | 38 (1.9%) | 0.692 |
